# Potentiating Effect of Beauvericin on Colistin, a Last Resort Antibiotic in Multidrug-Resistant *Pseudomonas aeruginosa* Strains

**DOI:** 10.3390/antibiotics15070631

**Published:** 2026-06-23

**Authors:** Ákos Suhajda, Mátyás Cserháti, Judit Háhn, Edit Kaszab, Melinda Fózer, Csilla Krifaton, Renáta Ábrahám, Balázs Kriszt

**Affiliations:** 1 Department of Molecular Ecology, Institute of Aquaculture and Environmental Safety, Hungarian University of Agriculture and Life Sciences (MATE), Páter Károly utca 1, 2100 Gödöllő, Hungary; suhajda.akos@uni-mate.hu (Á.S.); cserhati.matyas@uni-mate.hu (M.C.); abraham.renata@uni-mate.hu (R.Á.); 2 Department of Environmental Safety, Institute of Aquaculture and Environmental Safety, Hungarian University of Agriculture and Life Sciences (MATE), Páter Károly utca 1, 2100 Gödöllő, Hungary; kaszab.edit@uni-mate.hu (E.K.); fozer.melinda@phd.uni-mate.hu (M.F.); kriszt.balazs@uni-mate.hu (B.K.); 3 Department of Environmental Toxicity, Institute of Aquaculture and Environmental Safety, Hungarian University of Agriculture and Life Sciences (MATE), Páter Károly utca 1, 2100 Gödöllő, Hungary; cserhati.csilla@uni-mate.hu

**Keywords:** antibiotic resistance, colistin, beauvericin, *P. aeruginosa*, One Health perspective, ESKAPE pathogen

## Abstract

**Background**: The global emergence of antibiotic resistance highlights the urgent need for novel therapeutic strategies, including adjuvants and potentiating compounds, against multidrug-resistant bacteria. *Pseudomonas aeruginosa* is classified by the World Health Organization (WHO) as a critical priority pathogen due to its high resistance potential and its ability to cause severe nosocomial infections. Beauvericin (BEA), a frequently detected mycotoxin, has been reported to exhibit various bioactive properties, including potential antibacterial and potentiating effects. **Methods**: The interaction between BEA and a last-resort antibiotic, colistin (COL), was evaluated in seven multidrug-resistant *P. aeruginosa* isolates using a microplate-based growth assay after preliminary MIC tests. **Results**: BEA at non-inhibitory concentrations (2.5–10 µg/mL) significantly enhanced the antibacterial activity of COL (1 and 2 µg/mL) in six out of seven isolates, resulting in a marked reduction in residual bacterial growth. **Conclusions**: BEA exhibited no measurable antibacterial activity at the concentrations used in the combination experiments but acted as a strain-dependent potentiator of colistin activity against multidrug-resistant *P. aeruginosa*. The observed enhancement of colistin-mediated growth inhibition supports the potential of BEA as an antibiotic adjuvant at clinically relevant colistin concentrations and provides a basis for further mechanistic investigation.

## 1. Introduction

Antibiotic resistance is a globally emerging threat that poses a significant challenge to public health and animal welfare. Bacteria that have developed resistance to previously effective antibiotics are responsible for a large number of fatalities worldwide. Moreover, bacterial resistance is projected to become one of the leading causes of mortality by 2050, while the development of new therapeutic agents is substantially slower [1]. The World Health Organization (WHO) listed the ESKAPE (*Enterococcus faecium*, *Staphylococcus aureus*, *Klebsiella pneumoniae*, *Acinetobacter baumannii*, *Pseudomonas aeruginosa* and *Enterobacter* spp.) pathogens, which are identified as top-priority infectious microbes that can easily develop critical multidrug resistance against antibiotics [2].

*P. aeruginosa* (PAE) is a ubiquitous Gram-negative bacterium known to cause severe diseases like bacteraemia, pneumonia, intra-abdominal, gastrointestinal, skin and soft tissue and bloodstream infections, and often fatal nosocomial diseases in immunocompromised patients [3,4,5]. PAE infections are the leading cause of ventilator-associated pneumonia, and are among the most common causes of catheter-associated urinary tract infections [3]. Due to its exceptional metabolic versatility, *P. aeruginosa* can colonize and adapt to a broad spectrum of ecological niches, including water, wastewater, sediments, animal feces, compost, soil, and, especially, hydrocarbon-contaminated soils [6,7,8]. Although environmental and clinical isolates may differ in virulence and antibiotic resistance profiles, environmental PAE strains may pose similar health risks as clinical ones [9]. Furthermore, environmental pollutants (non-antibiotic compounds), including pesticides, have been shown to induce phenotypic antibiotic resistance in PAE isolates; therefore, environmental isolates may be exposed to more diverse resistance-inducing xenobiotics [10]. This bacterial species can develop extensive resistance to antibiotics after prolonged antibiotic therapy [5], partly due to its ability of utilizing multiple efflux pumps (such as MexAB-OprM or MexGHI-OpmD), antibiotic-modifying enzymes and biofilm-forming ability [11]. These adaptive strategies enable PAE to develop resistance against multiple classes of antibiotics. Therefore, in response to this growing threat, the WHO has established a classification system (AWaRe) to guide the appropriate use of antibacterial agents and categorize the antibiotics into Access, Watch and Reserve classes. According to these categories, Access antibiotics are the widely available treatment against the most common infections, with a narrow spectrum of activity and lower potential for the selection of antimicrobial resistance. The Watch group includes antibiotics with an increased potential for the selection of antimicrobial resistance; the usage of these antibiotics should be carefully monitored and typically reserved for use in hospital facility settings. Finally, the Reserve group contains the last-resort antibiotics that are used as a treatment for infections caused by multidrug-resistant bacteria [12].

Colistin (COL), also known as Polymyxin-E, is a non-ribosomal polypeptide antibiotic consisting of a seven-membered cyclic ring and a tripeptide side chain attached to a short fatty acid [13]. COL exerts its antibacterial activity against Gram-negative bacteria, as it can interact with the anionic component of lipopolysaccharide (lipid A) in the outer membrane, which is stabilized by divalent cations such as Mg^2+^ and Ca^2+^. The interaction of COL with the anionic lipid A layer leads to destabilization of the LPS layer, resulting in outer-membrane instability and ultimately cell death [13]. It was first isolated in 1947 by Koyama and colleagues from *Bacillus polymyxa subs. colistinus* and has been used in clinical settings since 1959 to treat various infections [14]; however, its clinical use was subsequently restricted due to reported nephrotoxicity in the 1970s [15]. In order to minimize the risk of nephrotoxicity, Landersdorfer and Nation [16] suggested that a plasma COL concentration of 2 µg/mL could be a reasonable target value for isolates characterized by a minimal inhibitory concentration (MIC) lower than 1 µg/mL [16]. According to the 2016 guidelines of the European Committee on Antimicrobial Susceptibility Testing (EU-CAST), the susceptibility breakpoint for colistin against *P. aeruginosa* was defined as a minimum inhibitory concentration (MIC) of 4 µg/mL. This breakpoint was established based on microbiological data, pharmacokinetic and pharmacodynamic (PK/PD) considerations, and available clinical outcome data. Previously, colistin has been a frequently used antibiotic in veterinary medicine as well. Although its general use is restricted nowadays, considering that it is a Reserve antibiotic according to the AWaRe classification, plasmid-mediated mobile colistin-resistance genes appearing in environmental bacterial strains (e.g., *E. coli*) e may pose a serious threat to human and animal health [17].

The One Health perspective is an interdisciplinary approach that recognizes human health, animal health and the environment as interdependent domains. It encompasses all ecological niches in which the off-target antimicrobial effects of environmental contaminants may contribute to the emergence and persistence of antibiotic-resistant microorganisms, thereby serving as potential reservoirs for resistance. In this context, agricultural settings (e.g., animal manure and livestock production) and wastewater treatment plants are considered major environmental reservoirs and potential sources of antibiotic-resistant bacteria (ARBs) that can infect both humans and animals.

Consequently, global efforts have been directed toward identifying new effective substances or combination therapies to combat bacteria that are developing resistance. Secondary metabolites of fungal origin are a major source of bioactive compounds that can have antibiotic properties. In addition to the many fungal-derived antibiotics in current use (penicillin, cephalosporins), there are plenty of secondary metabolites that have been described as potential targets of antibiotic discovery [18].

Besides the compounds characterized by certain beneficial properties for mankind (e.g., antibiotics), there are plenty of other compounds, such as mycotoxins, that can pose significant food safety risks. Beauvericin (BEA) is a naturally occurring cyclic-hexadepsipeptide and according to the World Mycotoxin Survey, it was the second most frequently detected mycotoxin in 2024 and the fourth in 2025 [19,20,21]. Despite its widespread occurrence, BEA—as an emerging mycotoxin—is not currently regulated under food and feed safety legislation. According to the EFSA panel on Contaminants in the Food Chain [22], BEA possibly does not pose a significant risk for human or animal health and is devoid of genotoxic potential [22]. Despite its classification as a mycotoxin, BEA has attracted considerable research interest recently, as shown by many promising bioactive properties, suggesting it as a potential candidate for pesticide and medicine research, as its antiviral, antibacterial, antifungal and insecticidal effects have been demonstrated [19,23,24].

The main potential benefit of BEA may lie in its potentiating effect, which has been observed in several cases. The presence of BEA can increase the cellular uptake of different chemicals, including antibiotic agents. Shekhar-Guturja and colleagues [25] demonstrated that BEA helps overcome azole fungicide resistance in *Candida glabrata* and *C. albicans* by inhibiting the ABC transporter systems. A similar study conducted by Tong et al. [26] described BEA as a drug efflux pump modulator. Another study carried out by Zhang et al. [27] demonstrated that a mixture containing 0.5 µg/mL BEA and 0.5 µg/mL ketoconazole fungicide has better efficacy against *Candida parapsilosis* than 50 µg/mL ketoconazole alone [27]. Although a previous publication [19] also proposed to investigate the effects of BEA on ARBs, there is a paucity of literature on the effects of BEA in combination with antibiotics. Recent findings by Vásquez-Bonilla and colleagues [28] have contributed to filling this gap, particularly highlighting the synergistic or additive effect of BEA in combination with antibiotics against Gram-positive *Staphylococcus aureus* [28]. Although a few publications demonstrate the potentiating effect of BEA in combination with other compounds, no studies have yet investigated these effects in Gram-negative bacteria.

Therefore, the objective of this study was to determine the antibacterial effect of BEA on the *P. aeruginosa* type strain ATCC 10145^T^, a clinical strain (ATCC 27853) and five environmental isolates (ATCC 15442, P14, P43, P69 and P114). Our further objective was to determine whether non-inhibitory concentrations of beauvericin can modulate or potentiate the antibacterial activity of colistin in multidrug-resistant *P. aeruginosa* isolates.

## 2. Results

### 2.1. Solo Effect of BEA

**Figure 1:**
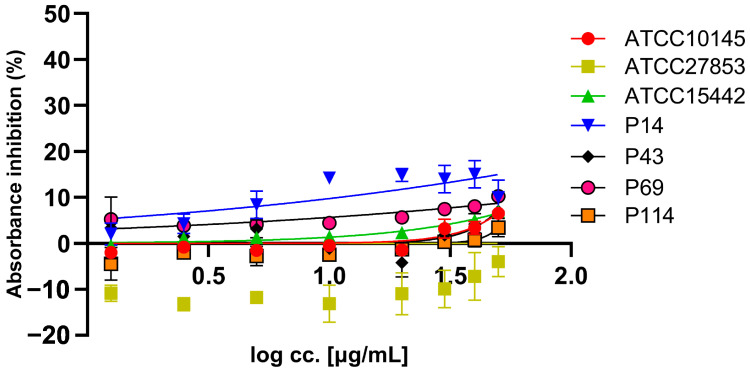
Concentration–response curves of BEA on the tested seven *P. aeruginosa* isolates. Absorbance inhibition (%) is shown in the axis Y, while the logarithm of concentration (µg/mL) is presented in axis X. Data were fitted using a four-parameter nonlinear regression model.

BEA is characterized by strong lipophilic properties (LogKow = 8.4, PubChem CID: 3007984), resulting in poor aqueous solubility. Consequently, a non-polar, non-volatile solvent with lower toxicity towards the investigated strains was required, leading to the selection of dimethyl sulfoxide (DMSO). According to our preliminary screen, the investigated strains fully tolerated DMSO concentrations up to approximately 1.25% (*v*/*v*, see Supplementary Figure S1A). Therefore, all experiments were conducted using a final DMSO concentration of 1% (*v*/*v*). Based on the results of the concentration–response analysis (Figure 1), BEA caused ≤10% absorbance inhibition at around 50 µg/mL concentrations, which indicates low toxicity at the tested concentrations. Net absorbance growth values representing the effects of BEA on *P. aeruginosa* strains with different initial absorbance values are shown in Supplementary Figure S1B.

### 2.2. Preliminary MIC Assay

After determining the inhibitory effect of BEA applied individually, BEA’s potentiating effect with seven antibiotics has been investigated on ATCC 27853, ATCC 10145^T^ and ATCC 15442 isolates (Table 1). According to these results, BEA increased the effect of COL and doripenem (DOR) in the investigated three *P. aeruginosa* isolates.

### 2.3. Microdilution Checkerboard Assay

According to our preliminary results obtained from the three tested isolates (ATCC 27853, ATCC 10145^T^ and ATCC 15442), BEA exhibited a potentiating effect on COL antibiotic. Therefore, to further investigate the interaction between COL and BEA, individual and combined effects were assessed in a microplate checkerboard assay with the three strains for preliminary testing (ATCC 27853, ATCC 10145^T^ and ATCC 15442 isolates), extended with another four (P14, P43, P69 and P114) environmental *P. aeruginosa* isolates. Two-way ANOVA revealed a significant interaction between treatment concentrations (*p* ≤ 0.05). Dunnett’s post hoc multiple-comparison test demonstrated that the effect of the COL + BEA combination varied depending on the concentrations, with higher concentrations generally resulting in stronger responses. Several concentration combinations reached statistical significance (*p* ≤ 0.05–0.001), as summarized in Table 2. Detailed statistical results, including the complete two-way ANOVA outputs and post hoc multiple-comparison analyses, are available in Supplementary Data S1.

#### Quantification of Colistin Potentiation by Beauvericin

Beauvericin significantly enhanced the inhibitory effect of colistin in a strain-dependent manner at non-toxic BEA concentrations (Table 2).

The most pronounced potentiating effects were observed at colistin concentrations of 1–2 µg/mL, corresponding to clinically relevant susceptibility breakpoints. In ATCC 27853, the addition of 2.5, 5 and 10 µg/mL BEA reduced the residual bacterial growth by 32.8%, 48.5%, and 69.8%, respectively, compared with colistin alone. Similarly, in ATCC 15442, 5 and 10 µg/mL BEA reduced residual growth by 65.8% and 68.3% at 1 µg/mL colistin, while at 2 µg/mL colistin, potentiation reached 56.4%, 96.4%, and 86.9% in the presence of 1.25, 2.5 and 5 µg/mL BEA, respectively. Among the environmental isolates, P14 showed strong potentiation, with residual growth reductions ranging from 47.8% to 84.2% at 2 µg/mL colistin and from 58.8% to 66.6% at 1 µg/mL colistin. Moderate but consistent potentiation was observed for P43, where residual growth was reduced by 18.6–49.6% depending on the colistin and BEA concentrations applied. Similarly, P69 exhibited reductions of 28.7–64.0% at 2 µg/mL colistin. The strongest response among the environmental isolates was observed in P114, where residual growth was reduced by 40.5% at 1 µg/mL colistin and by 40.7–94.0% at 2 µg/mL colistin. At 4 µg/mL colistin, the addition of 1.25 µg/mL BEA resulted in near-complete growth suppression. Despite its resistance to colistin, the ATCC 10145^T^ isolate exhibited increased susceptibility in the presence of BEA at elevated colistin concentrations. While no significant potentiation was detected at 1–2 µg/mL colistin, BEA reduced residual bacterial growth by approximately 20% and 65% at 4 and 8 µg/mL colistin, respectively.

Overall, significant potentiation was detected in six of the seven investigated MDR *P. aeruginosa* strains, indicating that sub-inhibitory concentrations of BEA can substantially enhance the antibacterial activity of colistin, although the magnitude of the effect was strongly strain-dependent.

Importantly, these effects were achieved at BEA concentrations that did not exert intrinsic antibacterial activity, supporting its role as a colistin potentiator rather than a second active antimicrobial agent.

## 3. Discussion

The extant literature suggests that BEA has antibacterial activity against a wide range of bacteria, including both Gram-negative (*E. coli*, *S. enterica*, *S. dysenteriae*, *Y. enterocolitica*, *P. aeruginosa*, *H. pylori*) and Gram-positive (*E. faecalis*, *L. monocytogenes*, *C. perfringens*, *S. aureus*) bacteria of clinical significance [23,29,30,31,32]. However, there are considerable differences in the bactericidal concentrations of BEA required for the inhibition of the tested isolates, even within the same species. Although previous studies have demonstrated that BEA may inhibit the growth of *P. aeruginosa* strains, the reported MIC values vary in a wide range, from 1 to more than 78.395 µg/mL [29,30,31]. The direct comparison of these findings is further complicated by the methodological differences between the studies. According to Meca et al. [29], 1000 ng of BEA dissolved in 10 mL of TSA agar (0.1 µg/mL concentration) inhibited the investigated *P. aeruginosa* isolates. Another study conducted by Olleik et al. [31] demonstrated that more than 100 µM of BEA (>78.395 µg/mL) can be estimated as a MIC value against the investigated *P. aeruginosa* isolates using a two-fold serial dilution test following the National Committee of Clinical Laboratory Standards (NCCLS, 1997) [31].

In our study, we investigated the MIC values of BEA in seven different *P. aeruginosa* strains (ATCC 27853, ATCC 10145^T^, ATCC 15442, P14, P43, P69 and P114), including both clinical and environmental MDR isolates. The interaction between BEA and the seven different *P. aeruginosa* isolates were investigated with a microplate checkerboard assay using absorbance measurements (550 nm) at 1% (*v*/*v*) DMSO concentration. According to the results, BEA shows no antibacterial effect (Figure 1) on the investigated isolates within the tested concentration range, up to 50 µg/mL. Due to the lipophilic nature of BEA, the maximum concentration that could be reliably tested was limited by its poor aqueous solubility. An increase in concentration beyond 12.5 µg/mL resulted in visible precipitation, whereas higher concentrations of DMSO (more than 1.25%) inhibited bacterial growth. In addition, growth measurements obtained at concentrations up to 50 µg/mL were corrected for the intrinsic absorbance of BEA in order to minimize optical interference. Accordingly, the lack of bactericidal activity under the applied conditions should be interpreted in the context of BEA’s limited solubility and the methodological limitations (opacity/interference) associated with the evaluations. The mechanism of action of BEA as an antibacterial agent is not fully understood currently, although Wang & Xu [19] suggest that BEA may not target the bacterial cell wall. Researchers suggest that, according to its ionophoric properties, BEA can form complexes with cations and may increase the permeability of biological membranes regardless of specific cell types [19], which may result in disruption of ion homeostasis in bacteria. Considering these findings, BEA may not be effective as an antibacterial agent against *P. aeruginosa* isolates in its naturally occurring chemical form, which corresponds with the study conducted by Olleik and colleagues [31].

BEA offers another possibility as it may increase the intracellular access of compounds with restricted membrane penetration. The initial observation of synergy between BEA and antibiotics was first reported by Vásquez-Bonilla in 2024 [28]. According to their study, BEA showed a synergistic effect in combination with oxacillin (mean Fractional Inhibitory Concentration Index (FICI) = 0.1578) and an additive effect with lincomycin (mean FICI = 0.507) against Gram-positive *Staphylococcus aureus* isolates. However, the same study showed that BEA can also have an antagonistic effect when combined with ciprofloxacin in Gram-positive *S. aureus* [28].

The current study provides the first insight into the combination of BEA with seven clinically relevant antibiotics (ceftazidime, cefepime, imipenem, doripenem, piperacillin, ciprofloxacin and colistin) on a Gram-negative bacterium, namely *P. aeruginosa*. The present study demonstrates a pronounced and strain-dependent potentiating effect of BEA on colistin activity against *P. aeruginosa*. Importantly, BEA was applied at concentrations that did not exert intrinsic antibacterial activity, thereby excluding direct toxicity as a sole antibacterial agent, but its potential may be realized as a true antibiotic adjuvant.

Since the MIC determination of BEA was not feasible due to the absence of detectable antibacterial activity against the tested isolates within the investigated concentration range, classical FICI-based interpretation was considered limited, as calculation of the FICI requires measurable MIC values for both compounds. In order to quantify the COL-enhancing effect of BEA, the percent residual growth inhibition effect calculation has been assessed. BEA substantially reduced residual bacterial growth under colistin exposure in a strain-dependent manner. Residual growth inhibition ranged from approximately 19% to 100%, with the strongest responses observed in ATCC 15442, P14, and P114. The results suggest that BEA-mediated modulation is highly strain-dependent and may involve adaptive responses, including biofilm formation, which could partially counteract the sensitizing effect of BEA. Overall, the observed effect should not be interpreted as a reversal of colistin resistance. Rather, the results demonstrate that sub-inhibitory concentrations of BEA can significantly enhance the antibacterial activity of colistin and further reduce residual bacterial growth in several MDR *P. aeruginosa* isolates, including at colistin concentrations corresponding to clinically relevant susceptibility breakpoints.

Interestingly, ATCC 10145^T^ exhibited only limited potentiation despite exposure to the same BEA concentrations. This observation further supports the strain-dependent nature of the interaction and may reflect isolate-specific physiological characteristics, such as differences in membrane composition, stress adaptation mechanisms, or efflux activity. Given that colistin primarily targets the outer membrane, although the underlying mechanism remains unclear, the observed effect may potentially be associated with subtle alterations in membrane permeability or another membrane-associated effect (such as membrane potential or membrane stability) induced by BEA, without independently compromising bacterial viability [23].

Future studies employing outer membrane permeability assays (e.g., NPN uptake) or membrane potential measurements (e.g., DiSC_3_(5) depolarization assays) may clarify whether BEA enhances colistin activity by facilitating antibiotic entry, destabilizing membrane integrity, or modulating proton motive force-dependent adaptive responses. Such mechanistic insight would further define BEA’s role as a membrane-modulating adjuvant rather than a conventional antimicrobial compound.

Collectively, these findings support the concept that sub-toxic natural products may modulate antibiotic responsiveness in multidrug-resistant *P. aeruginosa* and highlight the potential of BEA as a COL sensitizer at clinically relevant concentrations.

The effect of the combination of BEA and COL has not been investigated in previous studies; any conclusion regarding its potentiating effect remains to be speculative.

Based on previous studies, one possible explanation may involve the ionophoric properties of BEA and its ability to transport mono- and divalent cations across biological membranes [33]. Because colistin exerts its antibacterial activity through disruption of the outer membrane, which is stabilized by divalent cations such as Mg^2+^ and Ca^2+^ [34], alterations in cation transport or membrane-associated processes could potentially influence bacterial susceptibility to colistin. However, the present study did not investigate ion transport, membrane permeability, or cation homeostasis experimentally. Therefore, the proposed involvement of Ca^2+^-related processes should be regarded solely as a literature-based hypothesis requiring further experimental verification.

Alternative mechanisms may also contribute to the observed effect. Previous studies have also demonstrated that BEA can modulate xenobiotic susceptibility in eukaryotic systems, including fungi and protozoa, partly through inhibition of ABC transporters [24,25,26,27]. Whether a similar mechanism contributes to the observed potentiation in P. aeruginosa remains unknown. As efflux systems play an important role in multidrug resistance, modulation of bacterial efflux activity by BEA cannot be excluded and warrants further investigation. Future studies could help clarify the interactions between COL and BEA, extended with transcriptomic analysis to elucidate the deeper mechanisms of interaction with these compounds, which may also facilitate the development of more effective strategies against the increasing threat of antibiotic resistance.

The present study was limited to in vitro growth assays and did not distinguish between bacteriostatic and bactericidal effects. Furthermore, the observed responses were strain-dependent and included both potentiating and antagonistic interactions. Therefore, additional mechanistic and in vivo studies are required to clarify the biological basis and translational relevance of BEA-mediated modulation of colistin activity. Nevertheless, the reproducible potentiating effect observed in multiple MDR *P. aeruginosa* isolates identifies BEA as a promising candidate for further investigation as an antibiotic adjuvant.

## 4. Materials and Methods

### 4.1. Bacterial Strains

Four of the seven examined *P. aeruginosa* strains (P14, P43, P69 and P114) were isolated from hydrocarbon-contaminated sites and compost in Hungary and are deposited at −80 °C and in lyophilized form in the strain collection of the Department of Environmental Safety at the Hungarian University of Agriculture and Life Sciences (Gödöllő, Hungary). The species-level identification of these strains was performed with PCR amplification of the species-specific 16S rDNA regions V2 and V8 [6,35]. Three examined strains used in this study (ATCC 10145^T^ type strain, ATCC 27853 isolated from blood culture, and ATCC 15442 isolated from a water bottle in the animal room) were obtained from the National Collection of Agriculture and Industrial Microorganisms (NCAIM), Budapest, Hungary. The characteristics of the investigated isolates were previously described by Kaszab and colleagues [6,35,36]. All the investigated *P. aeruginosa* strains represented antibiotic-resistant phenotypes, and (excluding ATCC 15542^T^) met the MDR (multidrug resistance) criteria defined by Magiorakos and colleagues [37]. Details on the antibiotic resistance of the investigated isolates can be found in Supplementary Table S1.

### 4.2. Solo Antibacterial Effect of BEA

BEA (CAS: 26048-05-5, purity: 100%) was obtained from Fermentek Ltd. (Jerusalem, Israel). Stock solution was prepared in dimethyl-sulfoxide (DMSO, CAS: 67-85-5, purity: 99.9%, Fisher Scientific, Waltham, MA, USA) at a concentration of 5000 µg/mL. To assess the potential effects of BEA on *P. aeruginosa* strains, preliminary measurements were carried out to determine the maximum DMSO concentration tolerated by the strains. Two-fold dilution series of DMSO were created from 10 to 0.3 µL/mL final concentrations on 96-well, clear, U-shaped PS microtiter plates (Greiner Bio-One GmbH, Frickenhausen, Austria). In total, 10 µL of DMSO dilutions and 190 µL of LB media were distributed on the plates, then 50 µL of overnight *P. aeruginosa* inocula were adjusted to 0.6 optical density (at 600 nm, LB medium) and dispensed into the wells to reach a final volume of 250 µL. Optical density was measured at 550 nm using an absorbance reader (BioTek^®^ ELx800, Winooski, VT, USA) immediately after plate assembly and after 24 h of incubation (28 °C, 300 rpm).

In order to test the potential inhibitory effect of BEA, a microplate dilution assay was performed as described above on 96-well microplates using a two-fold dilution series of BEA from 50 to 2.4 × 10^−2^ µg/mL. According to the results of the preliminary tests, the final concentration of DMSO was adjusted to 1% (*v*/*v*). When using such a low concentration of organic solvent, opacity was observed at BEA concentrations of 12.5 µg/mL and above; therefore, to eliminate optical interference, the 24 h absorbance values were corrected using the corresponding 0 h absorbance values of each sample.

### 4.3. Preliminary MIC Assay

As a preliminary screen to test the combined effect of BEA with antibiotics in case of the ATCC 10145^T^, ATCC 27853 and ATCC 15442 bacterial cultures, an antibiotic resistance assay, with Liofilchem^®^ (Mast Diagnostica GmbH, Reinfeld, Germany) test strips, was carried out in order to determine the Minimal Inhibitory Concentrations (MIC values) of antibiotics in the presence of BEA. The use of well-characterized strains provided a standardized and reproducible framework for the preliminary assessment and served as the basis for the subsequent investigations involving a broader collection of isolates. Evaluation was performed according to the recommendations of the European Committee on Antimicrobial Susceptibility Testing (24 h of incubation at 35 °C). The overnight cultures of ATCC 10145^T^, ATCC 27853 and ATCC 15442 strains were centrifuged (4000× *g*, 4 °C, 30 min), then resuspended in sterile saline (0.85% NaCl) solution. The turbidity was adjusted to 0.5 McFarland with saline solution, then the strains were spread onto Mueller-Hinton agar (Merck Ltd., Darmstadt, Germany) plates without BEA and supplemented with 5 µg/mL of BEA. Then, test strips (ceftazidime, cefepime, imipenem, doripenem, piperacillin, ciprofloxacin, colistin) were placed onto the medium. After 24 h of incubation (35 °C), the differences in MIC values were calculated by subtracting the MICs obtained in the presence of BEA at 5 µg/mL from the MICs determined without BEA. Negative MIC values indicate a potential synergistic/additive/potentiating effect, 0 means indifference, and positive results demonstrate an antagonistic effect. Potentiating effects of BEA could be observed only in the case of colistin (COL).

### 4.4. Microdilution Checkerboard Assay

Based on the preliminary experiments, to further investigate the interaction between COL and BEA, the individual and combined effects of seven concentrations (16 to 0.25 µg/mL) of COL and ten concentrations (10 to 0.02 µg/mL) of BEA in two-fold dilution series were assessed in a microdilution checkerboard method [10] with the ATCC 10145^T^, ATCC 27853 and ATCC 15442 bacterial cultures extended with another 4 environmental *P. aeruginosa* isolates.

Microplate dilution assays were performed on 96-well, clear, U-shaped PS microplates (Greiner Bio-One GmbH, Frickenhausen, Austria). Dilutions of BEA and COL were prepared using 25% (*v*/*v*) DMSO in distilled water and distilled water, respectively. In total, 10 µL aliquots of the different concentrations of test materials were co-added to the x-and y-axes across the plate. Controls containing only BEA or COL were also prepared. Sterile LB medium was added to the wells to reach a volume of 200 µL, then 50 µL of overnight *P. aeruginosa* bacterial suspension in LB with an optical density of OD600  =  0.6  ±  0.02 was also dispensed into each well to adjust the final volume to 250 µL. In total, 190 µL LB supplemented with 50 µL bacterial suspension and with 10 µL of 25% DMSO (resulting in a final concentration of 1% (*v*/*v*) was used as a negative control. When using such a low concentration of organic solvent, slight opacity was observed at BEA concentrations of 12.5 µg/mL and above; therefore, to eliminate optical interference, the 24 h absorbance values were corrected using the corresponding 0 h absorbance values of each sample. Absorbance inhibition was calculated as described in [38] by comparing the corrected average bioluminescence values (CPS) of parallel samples to those of the negative control and was expressed as a percentage. The net OD values for each strain are shown in the Supplementary Data S2.

Assays were carried out in triplicate using freshly prepared solutions and bacterial suspensions. According to our previous experiments [10], to minimize excessive biomass sedimentation and improve the reproducibility of absorbance-based growth measurements, plates were incubated at 28 °C and at a speed of 300 rpm in a microplate shaker thermostat (PST-60HL-4, BioSan, Riga, Latvia). Absorbance was measured by an ELx800 microplate reader at 550 nm at the beginning of the incubation (0 h) and after 24 h of exposure.

### 4.5. Data Analysis

Statistical analyses were performed using GraphPad Prism 8 software, version 8.00 (GraphPad Software Inc., San Diego, CA, USA).

To determine the effects of DMSO and BEA, concentration–response curves were generated by plotting absorbance inhibition (%) against the logarithm of the concentration (µg/mL), then nonlinear regression analysis was performed using a four-parameter logistic model in order to determine inhibitory concentration values.

To calculate the differences between the COL and the COL + BEA combination, the absorbance values of the examined seven strains under co-exposure and their respective colistin control (samples containing bacterial suspension and colistin) were compared. The analysis of variance was performed with two-way ANOVA applied to Net growth absorbance data (Net growth = OD_24h_ − OD_0h_) originated from the microdilution checkerboard assay, followed by Dunnett’s multiple comparisons test. Differences with *p* ≤ 0.05 were considered significant.

### 4.6. Quantification of Colistin Potentiation by Beauvericin

Percent growth inhibition relative to the untreated growth control was calculated as:

$$I\;(\%)=100\times \frac{{\mathrm{Net}\;\mathrm{growth}}_{\mathrm{control}}-{\mathrm{Net}\;\mathrm{growth}}_{\mathrm{sample}}}{{\mathrm{Net}\;\mathrm{growth}}_{\mathrm{control}}}$$

where 0% indicates no inhibition compared to the growth control and 100% indicates complete suppression of net growth.

To quantify the potentiating (sensitizing) effect of BEA on COL independently of any intrinsic antibacterial activity of BEA, potentiation was expressed as the reduction in residual growth under COL exposure.

For each strain and COL concentration:

- ICOL denotes percent growth inhibition with COL alone (BEA = 0);
- ICOL + BEA denotes percent growth inhibition with the same COL concentration in the presence of BEA.

The absolute BEA-associated increase in inhibition was calculated as:

$$\Delta I=I_{\mathrm{COL}+\mathrm{BEA}}-I_{\mathrm{COL}}$$

Residual growth under COL alone corresponds to 100-I_“COL”. Therefore, the reduction in residual growth attributable to BEA (S%) was calculated as:

$$S\;(\%)=100\times \frac{I_{\mathrm{COL}+\mathrm{BEA}}-I_{\mathrm{COL}}}{100-I_{\mathrm{COL}}}$$

Positive S% values indicate potentiation, i.e., additional suppression of bacterial growth beyond the effect of COL alone.

Potentiation metrics (ΔI and S%) were calculated only for combinations in which BEA induced a statistically significant reduction in net growth compared with the corresponding COL-only condition (same COL concentration, BEA = 0).

## 5. Conclusions

Beauvericin (BEA) is a widely occurring mycotoxin with diverse biological activities and emerging potential as a bioactive modulator. The present study focuses on the solo and combined effects of BEA with seven medically important antibiotics against multidrug-resistant *P. aeruginosa* isolates. According to the results, BEA did not exhibit intrinsic antibacterial activity when tested alone. However, colistin (COL) was the only antibiotic for which BEA consistently enhanced antibacterial activity across multiple isolates. Although the magnitude of the effect varied considerably among isolates and was not observed uniformly across all tested strains, BEA significantly enhanced colistin activity and reduced residual bacterial growth in several MDR *P. aeruginosa* isolates. These findings indicate that BEA acts as a strain-dependent potentiator of colistin rather than a broadly effective antimicrobial adjuvant. Further investigations would be required to determine whether BEA-mediated potentiation can be exploited to broaden the possibilities of combating the emergence of multidrug-resistant Gram-negative bacteria. 

## Figures and Tables

**Table 1:** Baseline MIC values of the investigated antibiotics determined for the reference (wild-type) *P. aeruginosa* strain and the corresponding ΔMIC values in the presence of 5 µg/mL BEA. ΔMIC was calculated as MIC_BEA_ − MIC_wild type_. Negative values indicate decreased MICs (enhanced antibiotic activity), zero indicates no effect, and positive values indicate increased MICs (reduced antibiotic activity) in the presence of BEA.

Antibiotic Agent	ATCC 27853		ATCC 10145		ATCC 15442	
	MIC_WT_	ΔMIC	MIC_WT_	ΔMIC	MIC_WT_	ΔMIC
Ceftazidime	1	−0.5	3	0.0	2	4.0
Cefepime	8	−5.0	8	0.0	3	9.0
Imipenem	2	−0.5	3	5.0	0.75	2.25
Doripenem	1	−0.62	1	−0.5	0.5	−0.12
Piperacillin	4	−1.0	4	8.0	3	29.0
Ciprofloxacin	0.75	−0.5	0.38	−0.19	0.09	0.004
Colistin	6	−2.0	6	−3.0	4	−3.25

**Table 2:** Residual growth inhibition (RGI, %) and corresponding statistical parameters describing the potentiating effect of beauvericin (BEA) on colistin activity in the investigated *Pseudomonas aeruginosa* isolates. RGI values represent the percentage reduction in residual bacterial growth relative to the corresponding colistin-only treatment at the same colistin concentration. Mean differences were calculated from Net OD values (OD_24h_ − OD_0h_) between BEA-containing treatments and the corresponding colistin-only controls. Statistical significance was assessed using two-way ANOVA followed by Dunnett’s multiple comparisons test. Adjusted *p*-values and 95% confidence intervals (CI) are shown for all significant comparisons. NS indicates non-significant differences (*p* > 0.05).

Strain	COL cc.	BEA 1.25 µg/mL		BEA 2.5 µg/mL		BEA 5 µg/mL		BEA 10 µg/mL	
		RGI (%)	Mean Difference in Absorbance Values	RGI (%)	Mean Difference in Absorbance Values	RGI (%)	Mean Difference in Absorbance Values	RGI (%)	Mean Difference in Absorbance Values
ATCC 27853	1 µg/mL	NS	NS	32.8	0.3435	48.5	0.5073	69.8	0.73
					*p* < 0.0001		*p* < 0.0001		*p* < 0.0001
					CI = 0.1473 to 0.5397		CI = 0.3110 to 0.7035		CI = 0.5338 to 0.9262
ATCC 15442	1 µg/mL	NS	NS	NS	NS	65.8	0.681	68.6	0.9003
							*p* = 0.0001		*p* < 0.0001
							CI = 0.2628 to 1.099		CI = 0.4821 to 1.319
	2 µg/mL	57.9	6.6372	97.8	0.9078	86.3	1.164	100	1.047
			*p* = 0.0021		*p* < 0.0001		*p* < 0.0001		(*p* < 0.0001)
			CI = 0.1777 to 1.097		CI = 0.4484 to 1.367		CI = 0.7047 to 1.624		CI = 0.5870 to 1.506
ATCC 10145	4 µg/mL	NS	NS	NS	NS	NS	NS	19.6	0.281
									*p* = 0.0398
									CI = 0.008377 to 0.5536
	8 µg/mL	NS	NS	NS	NS	NS	NS	65	0.3753
									*p* = 0.0019
									CI = 0.1026 to 0.6479
P14	1 µg/mL	NS	NS	NS	NS	49.3	0.6077	55.5	0.6833
							*p* < 0.0001		(*p* < 0.0001)
							CI = 0.3470 to 0.8683		CI = 0.4227 to 0.9440
	2 µg/mL		0.3787	74	0.5883	84.1	0.6683	76.2	0.6057
		47.6	*p* = 0.0009		*p* < 0.0001		*p* < 0.0001		*p* < 0.0001
			CI = 0.1180 to 0.6393		CI = 0.3277 to 0.8490		CI = 0.4077 to 0.9290		CI = 0.3450 to 0.8663
P43	1 µg/mL	NS	NS	18.6	0.1237	25.9	0.1723	30	0.199
					*p* = 0.0422		*p* = 0.0012		*p* = 0.0001
					CI = 0.002774 to 0.2446		CI = 0.05144 to 0.2932		CI = 0.07811 to 0.3199
	2 µg/mL	29.6	0.138	32.5	0.1513	43.2	0.2013	49.6	0.231
			*p* = 0.0166		*p* = 0.0064		*p* < 0.0001		*p* < 0.0001
			CI = 0.01711 to 0.2589		CI = 0.03044 to 0.2722		CI = 0.08044 to 0.3222		CI = 0.1101 to 0.3519
	4 µg/mL	NS	NS	NS	NS	73.3	0.1417	64	0.1237
							*p* = 0.0129		*p* = 0.0422
							CI = 0.02077 to 0.2626		CI = 0.002774 to 0.2446
P69	2 µg/mL		0.2757	51.3	0.4953	63	0.608	63.4	0.612
		28.6	0.033		*p* < 0.0001		*p* < 0.0001		*p* < 0.0001
			CI = 0.01454 to 0.5368		CI = 0.2342 to 0.7565		CI = 0.3469 to 0.8691		CI = 0.3509 to 0.8731
P114	1 µg/mL	NS	NS	NS	NS	NS	NS		0.649
								40.7	*p* = 0.0095
									CI = 0.1113 to 1.187
	2 µg/mL		0.595	57.7	0.8443	85.4	1.25	94.1	1.377
		40.6	*p* = 0.0221		*p* = 0.0003		*p* < 0.0001		*p* < 0.0001
			CI = 0.05726 to 1.133		CI = 0.3066 to 1.382		CI = 0.7126 to 1.788		CI = 0.8396 to 1.915
	4 µg/mL		0.68		0.7007		0.6897		0.687
		99	*p* = 0.0057	100	*p* = 0.004	100	*p* = 0.0048	100	*p* = 0.005
			CI = 0.1423 to 1.218		CI = 0.1629 to 1.238		CI = 0.1519 to 1.227		CI = 0.1493 to 1.225

NS—not significant (*p* ≥ 0.05); RGI (%)—Residual Growth Inhibition (%).

## Data Availability

The data presented in this study are available within the article and its Supplementary Material. Further inquiries can be directed to the corresponding author.

## Supplementary Materials

The following supporting information can be downloaded at https://www.mdpi.com/article/10.3390/antibiotics15070631/s1: Supplementary Figure S1: (A) Effect of DMSO on bacterial growth expressed as absorbance inhibition (%). Values represent the mean inhibition calculated from all investigated Pseudomonas aeruginosa strains across the tested DMSO concentration range. Error bars indicate standard deviation. (B) Growth of the investigated P. aeruginosa strains in the presence of increasing concentrations of beauvericin (BEA), expressed as net optical density (Net OD; OD_24h_ − OD_0h_). No substantial growth inhibition was observed within the tested BEA concentration range, confirming the absence of intrinsic antibacterial activity under the applied experimental conditions; Supplementary Figure S2: Absorbance inhibition (%) of the seven investigated Pseudomonas aeruginosa isolates following combined exposure to colistin (COL) and beauvericin (BEA). For each isolate, bars represent the mean growth inhibition calculated from net OD values (OD_24h_ − OD_0h_) relative to the untreated bacterial control. Different bar colors indicate increasing COL concentrations, while the x-axis shows increasing BEA concentrations. Error bars indicate standard deviation. Asterisks denote statistically significant differences compared with the corresponding COL-only control at the same COL concentration, as determined by two-way ANOVA followed by Dunnett’s multiple comparisons test; Supplementary Data S1: Detailed statistical results of the two-way ANOVA outputs and Dunnett’s post hoc multiple-comparison analyses; Supplementary Data S2: Net OD values and absorbance inhibition values (%) for the investigated seven *Pseudomonas aeruginosa* strains after co-exposure of beauvericin and colistin in Microdilution Checkerboard Assays; Supplementary Table S1: Antibiotic resistance profile based on Minimal Inhibition Concentration (MIC) values and the origin of the investigated *Pseudomonas aeruginosa* strains.

## Abbreviations

The following abbreviations are used in this manuscript:

ARBs	Antibiotic-resistant bacteria
BEA	Beauvericin
COL	Colistin
DMSO	Dimethyl sulfoxide
EFSA	European Food Safety Authority
MDR	Multidrug-resistant
MIC	Minimal inhibitory concentration
PAE	*Pseudomonas aeruginosa*
WHO	World Health Organization
