# Supplementary material for: Potentiating Effect of Beauvericin on Colistin, a Last Resort Antibiotic in Multidrug-Resistant Pseudomonas aeruginosa Strains
Source: Antibiotics (Basel). 2026 Jun 23;15(7):631. doi: 10.3390/antibiotics15070631 (PMC13405934; doi:10.3390/antibiotics15070631)
Supplement: Supplementary file 1 [file antibiotics-15-00631-s001.zip › Supplementary Figure 1.pdf]

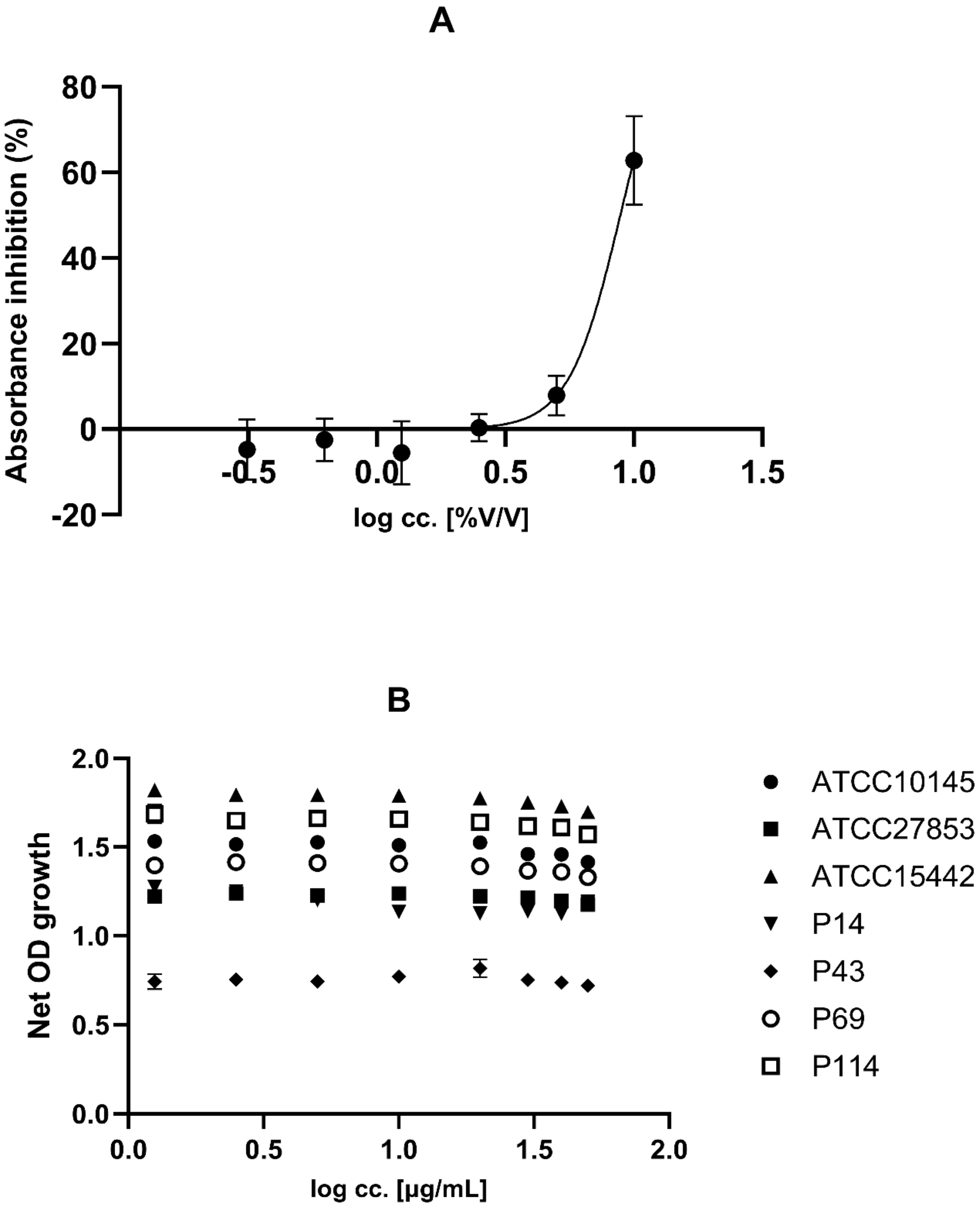

Supplementary Figure 1 - (A) Effect of DMSO on bacterial growth expressed as absorbance inhibition (%). Values represent the mean inhibition calculated from all investigated Pseudomonas aeruginosa strains across the tested DMSO concentration range. Error bars indicate standard deviation. **(B)** Growth of the investigated P. aeruginosa strains in the presence of increasing concentrations of beauvericin (BEA), expressed as net optical density (Net OD; $OD^{24h} - OD_{0h}$). No substantial growth inhibition was observed within the tested BEA concentration range, confirming the absence of intrinsic antibacterial activity under the applied experimental conditions
