# Supplementary material for: Potentiating Effect of Beauvericin on Colistin, a Last Resort Antibiotic in Multidrug-Resistant Pseudomonas aeruginosa Strains
Source: Antibiotics (Basel). 2026 Jun 23;15(7):631. doi: 10.3390/antibiotics15070631 (PMC13405934; doi:10.3390/antibiotics15070631)
Supplement: Supplementary file 1 [file antibiotics-15-00631-s001.zip › Supplementary tables Isolates properties.pdf]

		MIC values (ug/mL)															
		Cephalosporins					Penicillins		Carbapenems				Fluoroquinolones	Aminoglycosides			Miscellaneous agents
**Strain name**	**Origin**	**cefoperazone/sulbactam**	**Cefotaxime**	**Ceftazidime**	**Ceftriaxone**	**Cefepime**	**Piperacillin**	**Piperacillin/ tazobactam**	**Imipenem**	**Meropenem**	**Doripenem**	**Ciprofloxacin**	**Ofloxacin**	**Gentamicin**	**Amikacin**	**Tobramycin**	**Colistin**
ATCC 27853	Clinical (isolated from blood)	ND	ND	1	ND	8	4	ND	2	1.500	1.00	0.75	ND	1	ND	ND	6
ATCC 10145	Type strain, unknown source	ND	ND	3	ND	8	4	ND	3	1.500	1.00	0.38	ND	12	ND	ND	6
ATCC 15442	Water bottle in animal room	ND	ND	2	ND	3	3	ND	0.75	0.75	0.500	0.09	ND	1.500	ND	ND	4
P14	Hydrocarbon con-taminated soil	8	256	256	256	4	128	4	8	0.38	ND	0.125	2	8	8	2	1.5
P43	Hydrocarbon contaminated groundwater	16	256	64	256	24	256	4	>32	0.5	ND	0.125	2	256	3	>256	1.5
P69	Hydrocarbon contaminated soil	24	128	24	128	4	128	8	>32	0.5	ND	0.19	>32	2	6	1.5	2
P114	Compost	4	256	64	256	12	256	2	2	0.25	ND	0.125	0.75	4	6	1	1
	**EUCAST breakpoint https://www.eucast.org/bacteria/clinical-breakpoints-and-interpretation/clinical-breakpoint-tables/**	**ND**	**64**	**8**	**Insufficient data**	**8**	**16**	**16**	**4**	**2 (meningitis) 8 (other than meningitis)**	**2**	**0.5**	**4**	**8**	**16**	**2**	**4**

ND - no data
