# Supplementary figures and images for: Potentiating Effect of Beauvericin on Colistin, a Last Resort Antibiotic in Multidrug-Resistant Pseudomonas aeruginosa Strains
Source: Antibiotics (Basel). 2026 Jun 23;15(7):631. doi: 10.3390/antibiotics15070631 (PMC13405934; doi:10.3390/antibiotics15070631)

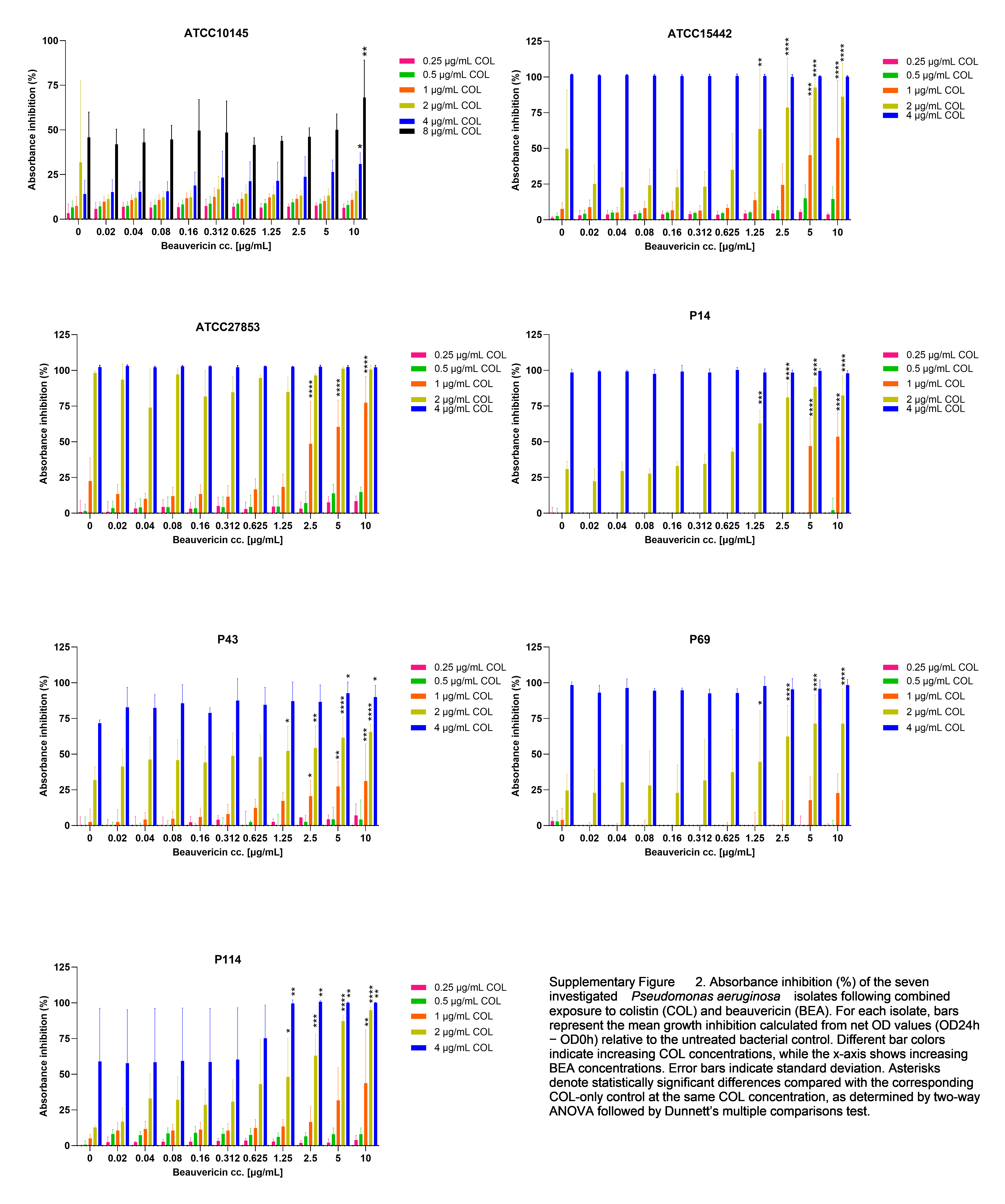

Supplement: Supplementary file 1 [file antibiotics-15-00631-s001.zip › Supplementary Figure2.tif]
